# Antimicrobial photodynamic therapy with fulleropyrrolidine: photoinactivation mechanism of *Staphylococcus aureus*, in vitro and in vivo studies

**DOI:** 10.1007/s00253-015-6539-8

**Published:** 2015-03-29

**Authors:** Mariusz Grinholc, Joanna Nakonieczna, Grzegorz Fila, Aleksandra Taraszkiewicz, Anna Kawiak, Grzegorz Szewczyk, Tadeusz Sarna, Lothar Lilge, Krzysztof P. Bielawski

**Affiliations:** 1 Laboratory of Molecular Diagnostics, Department of Biotechnology, Intercollegiate Faculty of Biotechnology, University of Gdansk and Medical University of Gdansk, Kladki 24, 80-822 Gdansk, Poland; 2 Department of Biotechnology, Division of Plant Protection and Biotechnology, Intercollegiate Faculty of Biotechnology, University of Gdansk and Medical University of Gdansk, Kladki 24, 80-822 Gdansk, Poland; 3 Laboratory of Human Physiology, Medical University of Gdansk, Tuwima 15, 80-210 Gdansk, Poland; 4 Department of Biophysics, Faculty of Biochemistry, Biophysics, and Biotechnology, Jagiellonian University, Gronostajowa 7, 30-387 Kraków, Poland; 5 Department of Medical Biophysics, Princess Margaret Cancer Centre/University of Toronto, 101 College Street 15-301, Toronto, ON M5G 1 L7 Canada

**Keywords:** Cytotoxicity, Fulleropyrrolidine, Mouse model, Photoinactivation, *Staphylococcus aureus*, Wound infection

## Abstract

A family of *N*-methylpyrrolidinium fullerene iodide salts has been intensively studied to determine their applicability in antimicrobial photodynamic therapy (APDT). This study examined in vitro the efficacy of a C_60_ fullerene functionalized with one methylpyrrolidinium group to kill upon irradiation with white light gram-negative and gram-positive bacteria, as well as fungal cells, and the corresponding mechanism of the fullerene bactericidal action. The in vitro studies revealed that the high antistaphylococcal efficacy of functionalized fullerene could be linked to their ability to photogenerate singlet oxygen and superoxide anion. Following *Staphylococcus aureus* photoinactivation, no modifications of its genomic DNA were detected. In contrast, photodamage of the cell envelope seemed to be a dominant mechanism of bactericidal action. In in vivo studies, a 2 log_10_ reduction in the average bioluminescent radiance between treated and non-treated mice was reached. One day post APDT treatment, moist and abundant growth of bacteria could be observed on wounds of non-fulleropyrrolidine and dark control mice. APDT-treated wounds stayed visibly clear up to the third day. Moreover, cytotoxicity test on human dermal keratinocytes revealed great safety of using the sensitizer toward eukaryotic cells. These data indicate potential application of functionalized fullerene as antistaphylococcal sensitizer for superficial infections.

## Introduction

Fullerene reactivity and applications have been studied since their discovery in 1985 (Kroto et al. 1985). However, the lack of solubility in biological friendly environments is the major obstacle for the development and clinical translation of this field. Thus, various functionalizations were and are being exploited to get more soluble compounds capable to interact with biological systems. The main efforts are focusing on extending the applications of fullerene and its derivatives. Antiviral and antibacterial activity as well as DNA cleavage are just a few aspects considered as potential fullerene applications, combined with the use of these compounds as drug delivery vehicle and gene therapy delivery vector (Marchesan et al. 2005; Mizuno et al. 2011; Tang et al. 2007; Yamakoshi et al. 1999; Isobe et al. 2006; Zakharian et al. 2005). In addition, fullerene C_60_ can induce radical production upon photoirradiation. The light radiation excites C_60_ from the ground state to ^1^C_60_, a short-lived species readily converted to the long-lived ^3^C_60_. The latter can transfer energy to molecular oxygen, if present, going back to the ground state. In this way, toxic ^1^O_2_ is generated. Moreover, fullerene in singlet and triplet states have been shown to be easily reduced to the corresponding C_60_ anions by electron transfer. All the reactive species can damage biomolecules such as lipids, proteins, and nucleic acids, which are common targets in photodynamic therapy. Furthermore, fullerenes absorb visible light and have a high triplet yield indicating their potential in photodynamic therapy (Castano et al. 2004). Fullerene showed antibacterial activity, which can be attributed to different interactions of C_60_ with biomolecules (Da et al. 1996). In fact, there is a possibility to induce cell membrane disruption or DNA cleavage. The fullerene hydrophobic surface can easily interact with membrane lipids and intercalate into them. When the cell damage is induced by reactive species other than singlet oxygen, type I mechanism takes place, while type II mechanism is considered when the damage is directly attributable to ^1^O_2_. Thus, fullerenes, and particularly, their soluble functionalized derivatives, are able to act as photosensitizers (PS) in photodynamic therapy (PDT). Moreover, recent studies indicate that fullerene functionalization with methylpyrrolidinium groups results in effective broad-spectrum antimicrobial PS that can broaden its application to antimicrobial photodynamic therapy (APDT).

Previous studies have shown that cationic fullerene derivatives, and in particular a family of *N*-methylpyrrolidinium fullerene iodide salts, are effective photosensitizers against numerous gram-positive and gram-negative bacteria; nevertheless, limited reports concerning its mechanism of bacterial photoinactivation as well as cytotoxicity toward eukaryotic cells are available (Dai et al. 2010; Dai et al. 2009; Lu et al. 2010). It is not yet clear if the photodynamic action implies the participation of superoxide and hydroxyl radicals (type I mechanism) or singlet oxygen (type II mechanism) and, also, if photoinactivation results from cell membrane or genomic DNA damage. Despite this, an efficacy of more than 3 log_10_ unit reduction in viable counts of bacterial and fungal cells in planktonic condition and a preferential selectivity for microbes over mammalian cells have been reported (Tegos et al. 2005). The cytotoxicity of these studied fullerene derivatives is often unknown; however, some reports concluded that C_60_ itself is non-toxic and is excreted from healthy eukaryotic cells and is also not being accumulated in any specific organ (Gharbi et al. 2005; Tabata et al. 1997). Therefore, in this work, we were interested in investigating bactericidal effect of mono-*N*-methylpyrrolidinium fullerene iodide salt toward gram-negative *Escherichia coli* and *Pseudomonas aeruginosa*, gram-positive *Staphylococcus aureus* and fungal cells, *Candida albicans*, and furthermore, in mechanistic aspects of the photodynamic action of this fullerene derivative against *S. aureus* cell planktonic suspension. The purpose was to evaluate possible photodynamic damages in the cells at the DNA and bacterial envelope level. These studies aim to provide a better understanding of the mechanism of the cellular death of *S. aureus* sensitized with functionalized fullerene. Furthermore, in this report, we describe mouse model of wound infected with bioluminescent derivative of methicillin-resistant *S. aureus* (MRSA) strain (Xen31) and topical application of fulleropyrrolidine to the infected site followed by green light illumination. With such designed study, we aimed at testing whether the high degree of light-mediated antimicrobial activity of functionalized fullerene in vitro translates also into an in vivo therapeutic effect.

Obtained results indicate that fulleropyrrolidine is a highly effective in vitro photosensitizer. Moreover, the very first screening tests reveal its safety and low toxicity against healthy human dermal cells. It acts generally through disrupting of bacterial envelope integrity and not DNA damage. In vivo studies indicated that the APDT treatment using fulleropyrrolidine iodide salt is beneficial in case of *S. aureus*-infected wounds; however, the effect is periodic and followed with bacterial regrowth.

## Materials and methods

### Cationic C_60_ fullerene derivative

**Fig. 1:**
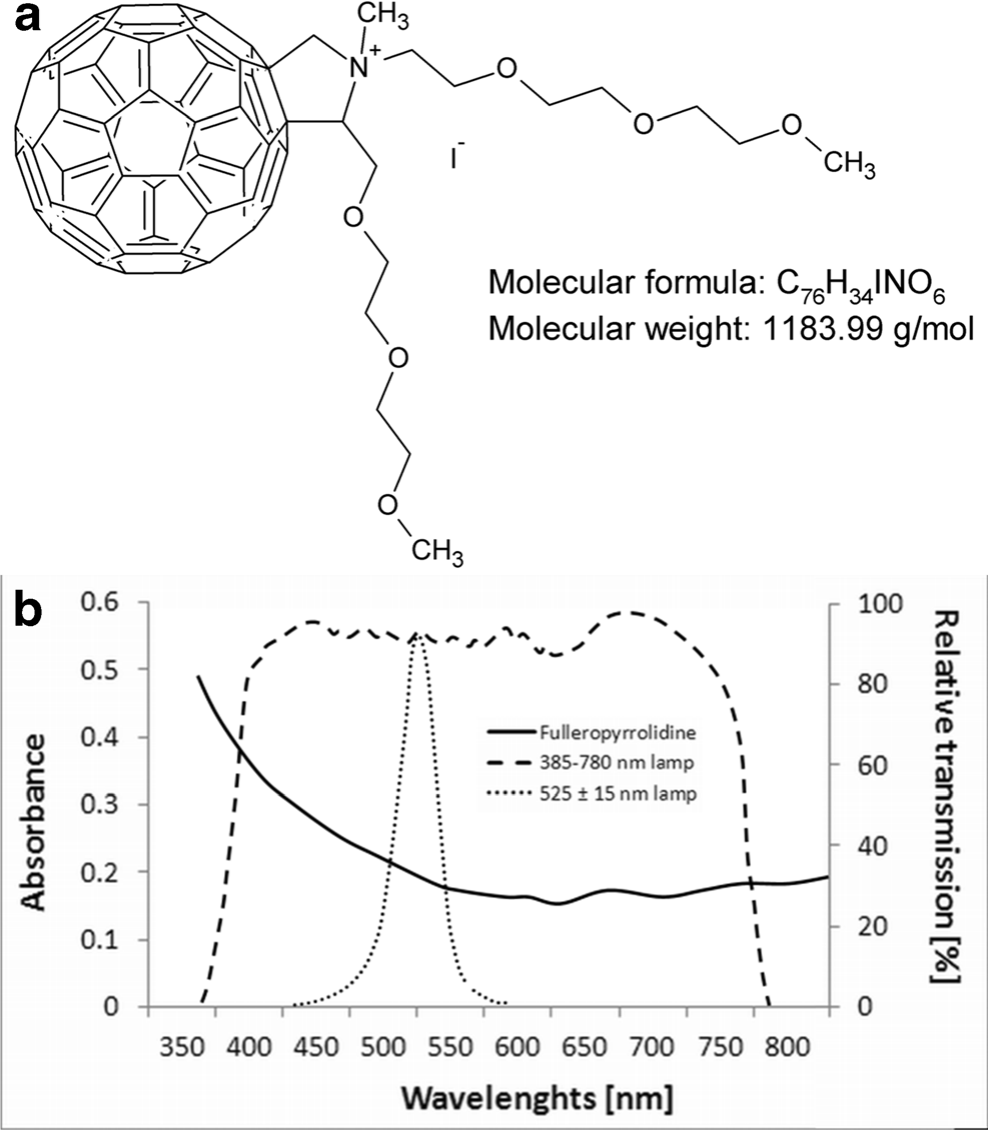
Photochemistry of fulleropyrrolidine. **a** Chemical structure of *N*-methylpyrrolidinium fullerene iodide salts. **b** Absorption spectrum of the sensitizer in solution of water and DMSO (*v*/*v*, 9:1) (10 μM) and output spectrum of the light sources

The synthesis, purification, and characterization of *N*-methylpyrrolidinium fullerene iodide salts by mass spectrometry (LR-MS, Agilent Technologies) was performed by ProChimia (Poland) according to previously described protocols (Da Ros et al. 1998). The compound was an analytically pure mixture of two diastereoisomers. The compound was maintained in the dark at 1 mM concentration in dimethylsulfoxide/ddH_2_O solution (*v*/*v*, 1:9) and diluted for in vitro or in vivo application. The structure is shown in Fig. 1, together with the visible absorption spectrum and spectral output of the white and green light lamp.

### Time-resolved spectroscopic detection of ^1^O_2_

Time-resolved luminescence of ^1^O_2_, in selected solvents of different polarity—acetonitrile, D_2_O, and 0.7 % Triton X-100 micelles in D_2_O—was measured at 1270 nm. Solutions of *N*-methylpyrrolidinium fullerene iodide salts in 1-cm fluorescence cuvette (QA-1000, HelmaOptik) were excited with 340 nm pulses (3.6 ns duration) generated by a fully tunable Nd:YAG laser (Expla NT242, Expla Vilnius, Lithuania), equipped with a spectral filtering accessory for improved spectral purity of pulses. At the selected wavelength, the laser system delivered 30 μJ pulses at 1 kHz repetition rate. Near-infrared luminescence was measured perpendicularly to the excitation beam in a photon counting mode using a thermoelectric-cooled NIR PMT module (Model H10330-45, Hamamatsu, Japan) equipped with a 1100-nm cutoff filter and additional selected narrow-band filters (NB series, NDC Infrared Engineering LTD, Maldon, UK). A computer-mounted PCI-board multichannel scaler was used for data acquisition (NanoHarp 250, PicoQuant GmbH, Berlin, Germany). First-order luminescence decay fitting by the Levenberg–Marquardt algorithm was performed employing a custom-written software.

### Detection of oxygen radicals

EPR spin trapping was employed using 5,5-dimethyl-1-pyrroline *N*-oxide (DMPO) as a spin trap at concentration 10 mM. Samples in EPR quartz flat cells were irradiated in the resonant cavity with 540–740 nm (70 mW/cm^2^) light derived from a 300 W high pressure compact arc xenon lamp (Cermax, PE300CE–13FM/Module300W, PerkinElmer) equipped with a 5-cm water filter, heat reflecting hot mirror, cutoff filter blocking light below 390 nm, and long-pass filter transmitting light above 540 nm. EPR samples were run using 10 mW microwave power, 0.05 mT modulation amplitude, 8.0 mT scan width, and 10 s scan time. Typically, EPR data consisted of four accumulated EPR spectra. EPR measurements were carried out using a BrukerEMX–AA EPR spectrometer (BrukerBioSpin, Germany). Experimental EPR spectra were compared with simulated EPR spectra using a WinSIM (Version 0.98) program.

### Strains and culture conditions

For in vitro testing, the following microbial strains were used: *S. aureus* (MSSA reference strains 8325–4 and Newman, and MRSA Xen31), *P. aeruginosa* (ATCC 10145), *E. coli* (ATCC 25922), and *C. albicans* (ATCC 10123). For in vivo studies, the MRSA strain used was the stably bioluminescent MRSA Xen31 (Xenogen Corp., Alameda, CA), which was derived from the parental strain *S. aureus* ATCC 33591. The bioluminescent strain was transformed with a chromosomal copy of the modified *Photorhabdus luminescens* luxABCDE operon (Francis et al. 2001). Microbes were grown overnight in brain heart infusion (BHI) medium at 37 °C while shaking at 150 rpm.

### In vitro APDT

Microbial overnight cultures were centrifuged (1000 *× g*) for 3 min and then re-suspended in phosphate-buffered saline (PBS). A cell suspension consisting of 10^7^ colony forming unit (CFU)/ml for bacteria and 10^6^ CFU/mL for *C. albicans* was incubated with fullerene for 30 min at room temperature in the dark. Next, 100 μl aliquots of cell suspension were transferred to a 96-well plate. The highest concentration of DMSO used was below 0.1 %, and this did not cause any toxicity to the cells. Illumination was performed using a Q.Light PDT Lamp (b & p Shweiz AG, Rorschach, Switzerland) (ISO 9001 & EN 46001—CE 1275) (irradiance 267 mW/cm^2^). To obtain the reported fluence, the distance between the device and the samples was 3 cm and the spot area was 2 cm in diameter. The spatial profile of the beam emitted by the Q.Light PDT Lamp has a Gaussian distribution; therefore, there is 10–15 % higher energy level in the center of the beam. Therefore, the delivered light energy was determined using a light power meter (model LM1, CARL ZEISS, Jena, Germany) in the central spot area of 2 cm in diameter. To assure precise illumination of all samples, simultaneous irradiation was performed solely over four wells of microtiter plates fitted into the illumination spot area. The Q.Light PDT Lamp emits polarized light (polarization level 98 %) over a wavelength range of 385–780 nm. Samples were illuminated for 10 min to deliver 160 J/cm^2^. At the completion of the illumination period, without any further incubation, 10 μl aliquots were removed from illuminated and non-illuminated wells (cells consisted as a control, kept in 96-well plates covered with aluminum foil at room temperature for the duration of the illumination) and serially diluted 10-fold in PBS to give dilutions of 10^−1^ to 10^−4^ times the original concentrations, and 10 μl aliquots of each of the dilutions was streaked horizontally on square petri dishes with brain–heart infusion medium (BHI, BioMerieux, France) (Jett et al. 1997). Plates were streaked in triplicate and incubated for 24 h at 37 °C in the dark to allow colony formation. Control groups included cells that were not treated with fullerene or light and cells treated with light but not with fullerene. Survival fractions (SFs) were expressed as ratios of CFU of microbial cells treated with fullerene to CFU of non-treated microbes. Each experiment was performed three times for statistical analysis.

### Loss of 260 nm absorbing material

Cytosolic leakage of nucleic acids was determined by measuring the amount of 260 nm absorbing material (Chen & Cooper 2002). Overnight *S. aureus* cultures were diluted with fresh BHI medium to an optical density OD_600_ 0.05 and cultured at 37 °C with shaking (150 rpm) to mid-log growth phase (OD_600_ 0.7). Final cell suspension was centrifuged (1000 *× g*, 3 min), washed and re-suspended in PBS, treated with APDT (10 μM fullerene derivative, radiant exposure of 160 J/cm^2^ of white light), or incubated with fullerene in dark or treated only with light. The suspension was centrifuged for 10 min at 12,000 *× g*, and the resulting lysate was filtered using 0.22 mm filters to remove any remaining planktonic bacteria. The absorbance at 260 nm of the filtered lysate was recorded using NanoDrop (Thermo Scientific) UV–VIS spectrophotometer. Total cytosolic nucleic acid content was estimated by boiling an untreated suspension of bacteria for 30 min. Sterile PBS served as a blank. Experiments were performed three times for statistical analysis (Connell et al. 2013).

### Cell membrane integrity

For cell membrane studies, *S. aureus* in mid-log growth phase, centrifuged (1000 *× g*, 3 min), washed, and re-suspended in PBS, was exposed to APDT treatment or control conditions (treated with fullerene in dark, treated only with light and kept in dark). Additionally, non-treated bacteria were exposed to 0.01 % benzethonium chloride (BCl; cell membrane disruption positive control) for 1 h. Next, propidium iodide (PI; at a final concentration of 5 μg/ml) was placed in each sample, and the cells were incubated for an additional 30 min in the dark at room temperature. Following staining, the bacteria were centrifuged, washed, and re-suspended in 1 ml PBS. Sample fluorescence was read using an EnVision Multilabel Plate Reader (PerkinElmer) with 488/570 nm excitation and emission filters. Additionally, samples were stained with SYTOX Green (Molecular Probes) at the final concentration of 5 μM for 10 min in room temperature. Next, measuring of fluorescence signal of DNA-bound SYTOX Green was performed using an EnVision Multilabel Plate Reader (PerkinElmer) with 504/523 nm excitation and emission filters. Experiments were performed three times and analyzed statistically (Connell et al. 2013).

### Genomic DNA extraction and polymerase chain reaction

Cell suspension of *S. aureus* in mid-log growth phase in PBS was treated with 10 μM fullerenpyrrolidine as previously indicated. Culture tubes were irradiated for 10 min with visible light. As a positive control, *S. aureus* was treated with 100 μM TMPyP and illuminated with 40 J/cm^2^ of red light (630 nm). Post irradiation, the genomic DNA (10 ng) was extracted from the *S. aureus* cells using Extract *ME* DNA Bacteria purification kit (Blirt S.A.) and treated with endonuclease III (New England, BioLabs). Endonuclease III from *E. coli* acts as both *N*-glycosylase and an apurinic/apyrimidinic (AP) lyase. The *N*-glycosylase activity releases damaged pyrimidines from double-stranded DNA, generating a basic (AP site). The AP lyase activity of the enzyme cleaves 3′ to the AP site leaving a 5′ phosphate and a 3′ ring-opened sugar. This treatment allows visualization of all photoinduced damages of genomic DNA. Genomic DNA samples were analyzed by electrophoresis (0.8 % agarose gel, with ethidium bromide staining at 120 V). Moreover, the genomic DNA amplification of conserved bacterial regions, that is, 16S ribosomal RNA (rRNA), was performed using the primers 16S rRNA-F 5′-GCAAGCGTTATCCGGATTT-3′ and 16S rRNA-R 5′-CTTAATGATGGCAACTAAGC-3′. All reactions were performed with each primer (0.6 pmol), 1 ng of genomic DNA as template, 200 μM dNTPs, 3.13 mM MgCl_2_, 1× PCR buffer, and 0.75 U Taq DNA polymerase (ThermoScientific, Lithuania). Amplification program was conducted as follows: initial denaturation step at 95 °C for 5 min, followed by 25 cycles of denaturation at 94 °C for 30 s, annealing for 30 s at 60 °C, and extension at 72 °C for 30 s, followed by an extra cycle of annealing at 60 °C for 30 s and a final extension at 72 °C for 5 min. The PCR products were analyzed by electrophoresis on 1.5 % agarose gels, with ethidium bromide at 120 V (Al-Talib et al. 2009).

### Cytotoxicity and phototoxicity of functionalized fullerene

Cell culture: The human keratinocyte (HaCaT) cell line was cultured in Dulbecco’s modified Eagle’s medium (DMEM) supplemented with 4500 mg/l glucose, 10 % fetal bovine serum, 2 mM glutamine, 10,000 U penicillin, and 10 mg/ml streptomycin. Cultures were maintained in a humidified atmosphere containing 5 % CO_2_ at 37 °C. Cells (3 × 10^4^) were seeded into 96-well plates and allowed to adhere overnight. Fullerene was examined in the concentration range of 0–100 μM. Cells were incubated for 30 min at 37 °C in the dark; subsequently, the medium was discarded and replaced with new medium. Cells designated for phototoxicity analysis were irradiated for 10 min as indicated above with white light at an irradiance of 16 J/cm^2^/min. Cells were further incubated for 24 h, after which cell survival was determined with the (3-(4,5-dimethylthiazol-2-yl)-2,5-diphenyltetrazolium bromide (MTT) assay. Briefly, MTT (0.5 mg/ml) was added, and cells were incubated for 3 h at 37 °C. Cells were lysed with DMSO, and the absorbance of the resulting formazan solution was measured at 550 nm with a plate reader (Victor, 1420 multilabel counter).

### Animal experiments

All animal procedures were approved by Animal Care Committee (ACC) of the University Health Network’s Animal Resources Centre, Toronto, ON Canada. A total of 17 FVB/N luc iNOs mice of both sex, weighing 20–25 g, were used. Mice were housed under room temperature on 12-h day/night cycles. Mice were divided into five groups (four groups of three animals each and one group of five animals) as follows: non-infected mice (*n* = 3), non-treated mice [PS (−); light (−)] (*n* = 3), dark control assay [PS (+); light (−)] (*n* = 3), light control [PS (−); light (+)] (*n* = 3), and APDT assay [PS (+); light (+)] (*n* = 5). The day prior to the experiment, mice were anesthetized by isoflurane (4 % induction in an induction chamber and 1.5–2.5 % maintenance in air via nose cone) and shaved on the back and depilated with depilatory lotion. Next day, a small pinch of skin from shaved area was held by tweezers and cut on the top, to establish an excisional wound down to, but not through, the panniculus carnosus measuring approximately 5 × 5 mm. There was no visible bleeding within the wounds. Infection was carried out by applying 10 μl of a suspension of bacteria in PBS containing 10^7^ log-phase colony forming unit (CFU) of *S. aureus* Xen31. Next, the wound was covered by dressing (Tegaderm™) to avoid cross-contamination. Thirty-minute post bacterial inoculation, 10 μl of 30 μM functionalized fullerene was injected under the dressing by Hamilton syringe and spread over the infection site. A final concentration of fullerene of ~5 × 10^7^–7 × 10^7^ molecules per bacterium was used. Light was administered 30 min post photosensitizer administration. Illumination was performed with a custom-built high-power LED light source (Theralase™, Toronto, ON) emitting at 525 ± 15 nm and delivering an irradiance of 50 mW/cm^2^ to the skin surface. The dressing was light transparent at this wavelength as previously demonstrated (Lilge et al. 2000).

### Bioluminescence imaging

All images were taken using IVIS Spectrum imaging system (Caliper Life Sciences). Prior to the bioluminescence radiance quantification, mice were anesthetized using isoflurane (as described above). Mice were imaged at specific time points: after bacteria inoculation, before photosensitizer injection, after sensitizer administration, before light exposure, after illumination if applicable, and every 10 min post APDT up to 2–3 h. Additionally, bacterial luminescence from mouse wounds was recorded daily for up to 5 days or death/euthanasia as applicable. Effectiveness of APDT and bacterial cell killing was measured by the magnitude of the bioluminescent signal, defined as average radiance ([hv/s/cm^2^/sr]) and by visual evaluation of the wound’s appearance.

### Statistical methods

Means of in vitro survival fractions and fractions of bioluminescence remaining were analyzed with one-way analysis of variance (ANOVA). Any *p* values of less than 0.05 were considered significant.

## Results

### Photochemical studies

**Fig. 2:**
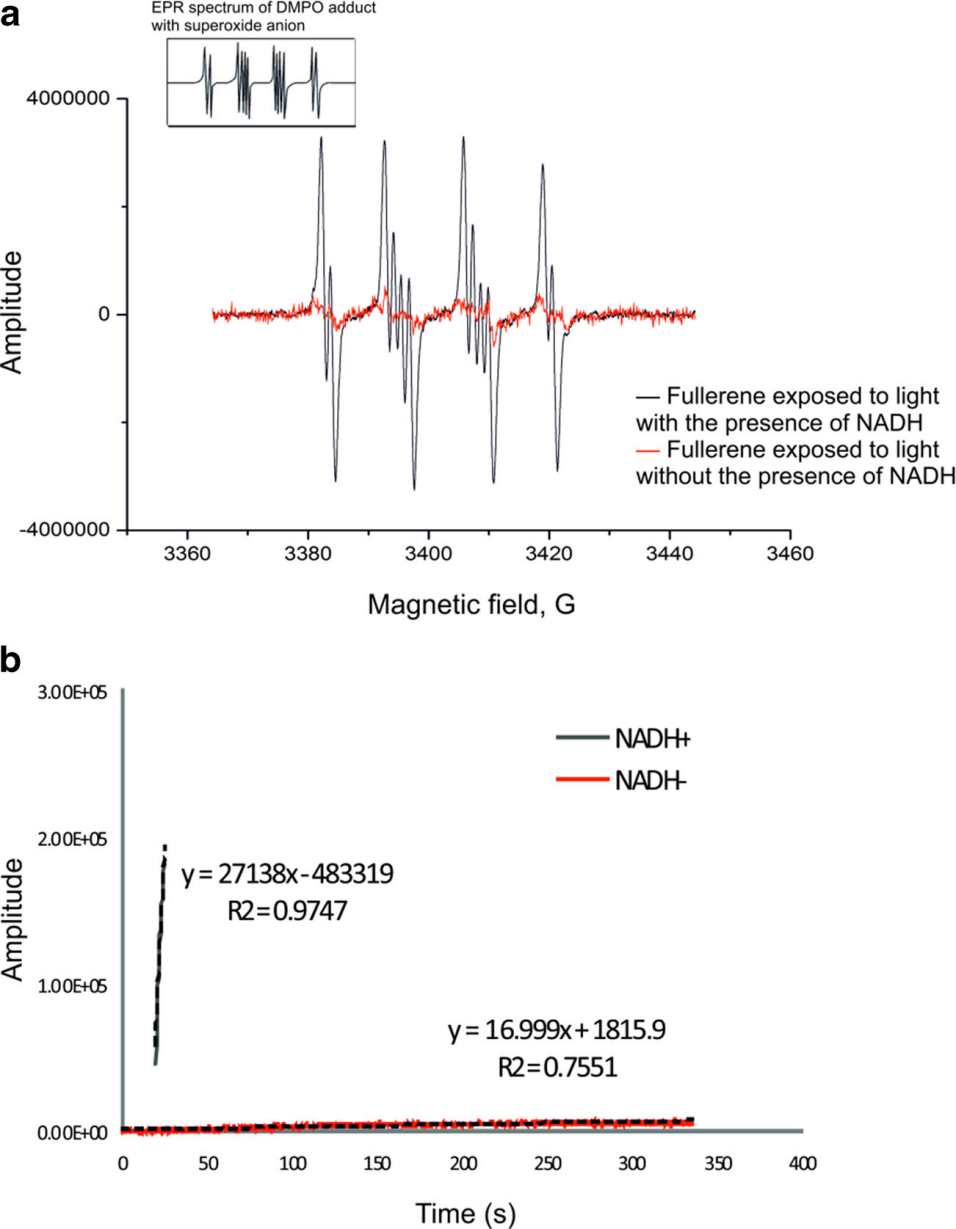
**a** EPR spectra of DMPO adducts arised after fullerene exposed to light. **b** Electron paramagnetic resonance (EPR) spin trapping results. Example of EPR spectrum of the DMPO spin adduct that evolved with irradiation time of the fullerene in the resonant cavity

To determine the mechanism of photodynamic action of fulleropyrrolidine, two independent methods were employed: direct measurements of ^1^O_2_ luminescence and spin trapping of free radicals. The quantum yield of singlet oxygen photogeneration in D_2_O was found to be very low, which confirmed results reported by other researchers that in aqueous solutions, even functionalized fullerenes C_60_ with one positively charged group were very inefficient mediators of type II photochemistry and produced only a negligible amount of singlet oxygen. Thus, the estimated quantum yield of singlet oxygen for the fullerene was 0.31 % ± 0.02, while that of rose bengal was 70 %. The measurements of time-resolved luminescence at 1270 nm were also carried out in a polar organic solvent and in micelles formed by a non-ionic detergent. In acetonitrile solution, after excitation at 340 nm, the photogeneration of singlet oxygen was comparable to that for rose bengal, which was estimated to be 83 % ± 10. Similarily, measurement of quantum yield of singlet oxygen in microenvironment formed by Triton X-100 micells revealed that the photogeneration of singlet oxygen by the fullerene was significant, with the corresponding quantum yield being 45.6 %, which was only 2-fold lower than that of rose bengal (83 % ± 10). In the second type of experiments, the formation of oxygen radicals was detected by EPR spin trapping using DMPO as a spin trap. Upon irradiation of the fullerene samples in DMSO/H_2_O (9:1) mixture, generation of the DMPO-superoxide anion adduct was observed (Fig. 2a). As evident from Fig. 2b, the rate of the spin adduct accumulation was dramatically increased in the presence of NADH as an electron donor (Fig. 2b).

### In vitro APDT

**Fig. 3:**
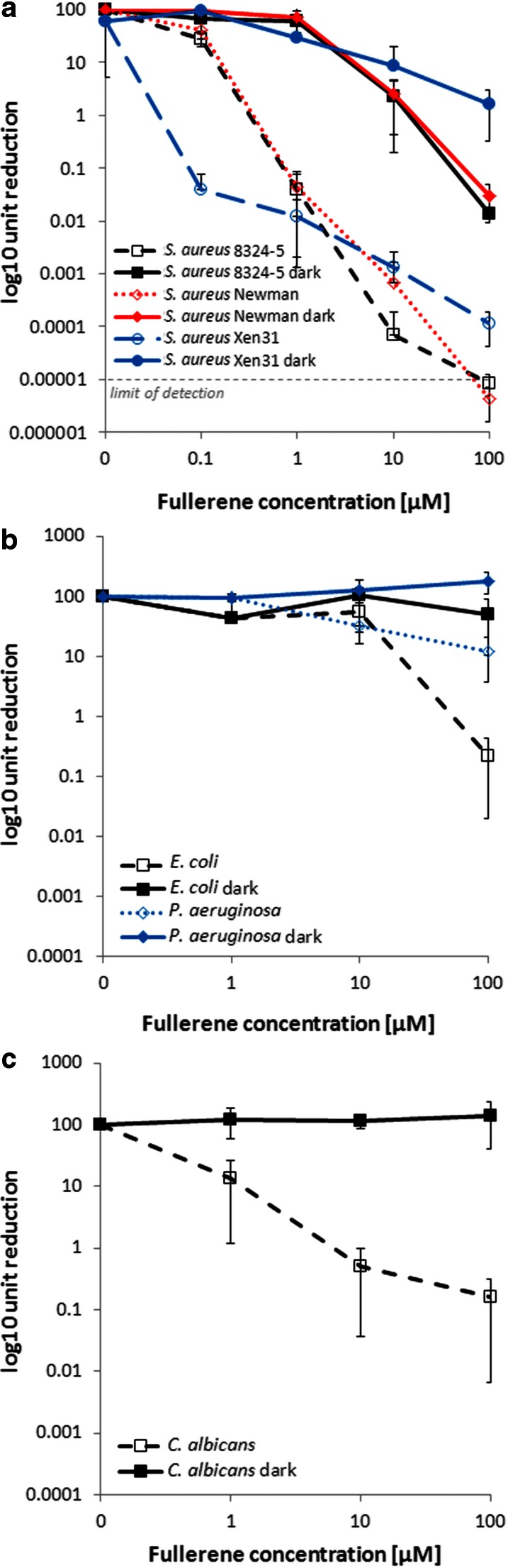
In vitro fullerene derivative photodynamic therapy. Microbial cell planktonic suspensions were incubated for 30 min with various concentrations of sensitizer and then illuminated or without 160 J/cm^2^ of 385–780 nm light. Values are means of three separate experiments, and *bars* represent one SD

The in vitro APDT bactericidal effect against two gram-negative species (*E. coli* and *P. aeruginosa*) as well as gram-positive *S. aureus* and *C. albicans* is presented in Fig. 3. The susceptibilities of four different microbial species were tested for increasing concentration of fulleropyrrolidine at constant radiant exposure. There was no dark toxicity of the sensitizer toward gram-negative and fungal species at studied concentrations (reaching 100 μM). In case of *S. aureus*, fulleropyrrolidine in the absence of light induced bacterial inactivation by 2 log_10_ unit reduction in viable counts at concentration of 10 μM. Inactivation of *S. aureus* was significant at 1 and 10 μM, leading to 3.5 and >6 log_10_ unit reduction, respectively. In the case of gram-negative species, concentration of 100 μM resulted in photoinactivation of 1 and 3 log_10_ unit reduction of *P. aeruginosa* and *E. coli*, respectively. Photokilling of *C. albicans* was concentration-dependent and reached 3 logs at 100 μM concentration. Light treatment, without sensitizer, did not exert inactivation of microbial cells; thus, its effect was not presented in Fig. 3.

### Photodynamic effect of fulleropyrrolidine on the leakage of intracellular DNA and cell membrane permeabilization

As bactericidal effect of the studied sensitizer was most pronounced in the case of *S. aureus* cells, *S. aureus* Newman was considered the best model for further mechanistic studies concerning analyzed fullerene derivative and in particular its effect on the cell membrane envelope. Moreover, this *S. aureus* strain is a wild-type strain with an intact outer and inner membrane.

**Fig. 4:**
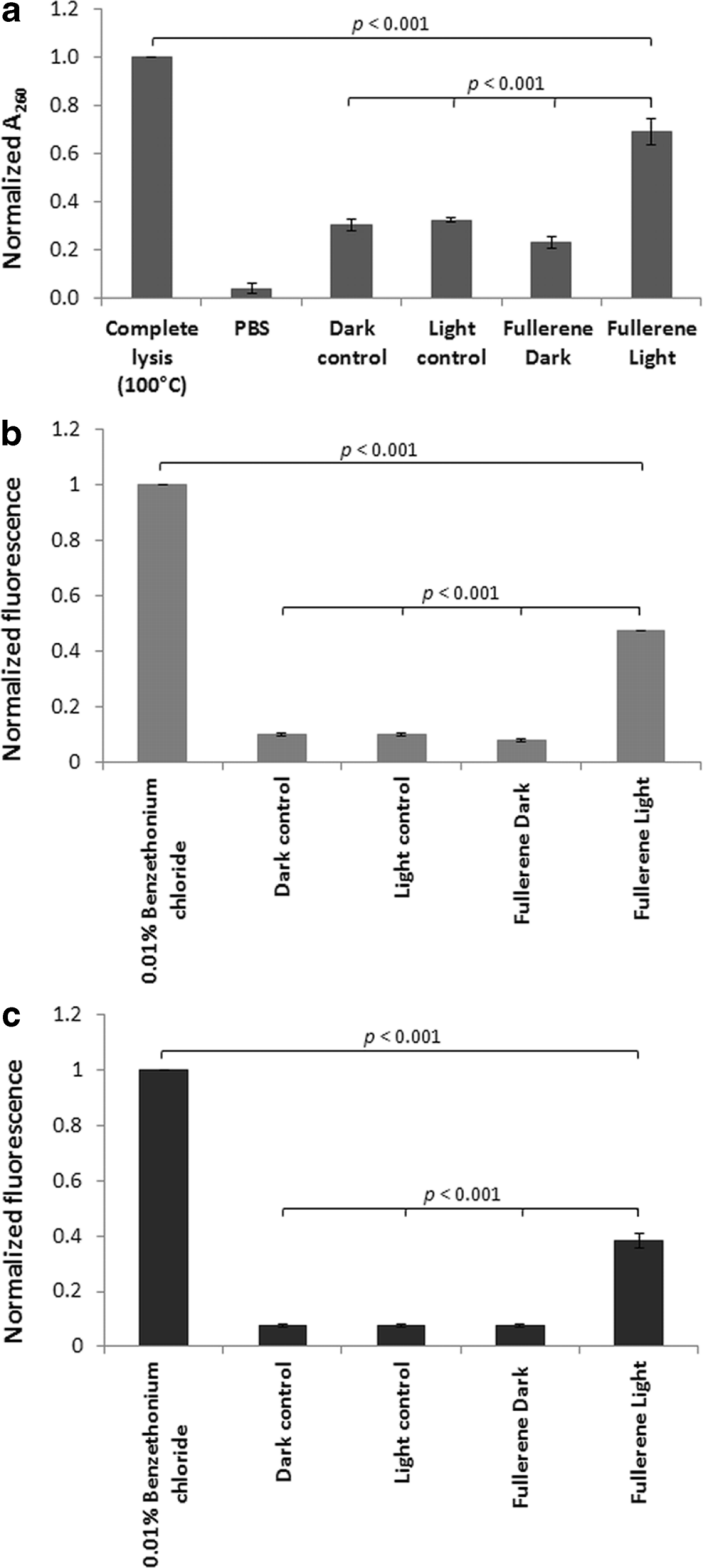
*S. aureus* cell membrane integrity. **a** Normalized A_260_ readings of cell culture supernatants after treatment. All normalized values are with respect to the same bacterial populations boiled for 30 min. **b** Total SYTOX Green fluorescence readings for the different groups. **c** Total propidium iodide fluorescence readings for the different groups. All groups were statistically significant compared with the negative and positive control and with each other. All normalized values are with respect to the same bacterial populations treated for 1 h with BCl (0.01 %)

To monitor bacterial membrane damage, the release of biopolymers was monitored by UV absorption at 260 nm. Supernatants from treated and control *S. aureus* suspensions were analyzed by UV spectroscopy as shown in Fig. 4a. The absorbance at 260 nm was significantly higher in the case of APDT-treated cells in comparison to control samples or cells treated solely with fullerene derivative in the absence of exciting photons. This suggests that membrane damage induced by fulleropyrrolidine is sufficient to produce release of higher molecular weight species.

Further, cell membrane integrity studies were conducted using SYTOX Green, and propidium iodide staining was applied (Fig. 4b, c). Fullerene-mediated APDT resulted in increased membrane permeability as indicated by increased fluorescence intensities of DNA-bound SYTOX Green (Fig. 4b). Additionally, propidium iodide uptake was increased in APDT-treated samples (Fig. 4c). In all cases, benzethonium chloride (0.01 %) was used as the positive control for membrane permeabilization.

### APDT effect on *S. aureus* genomic DNA

**Fig. 5:**
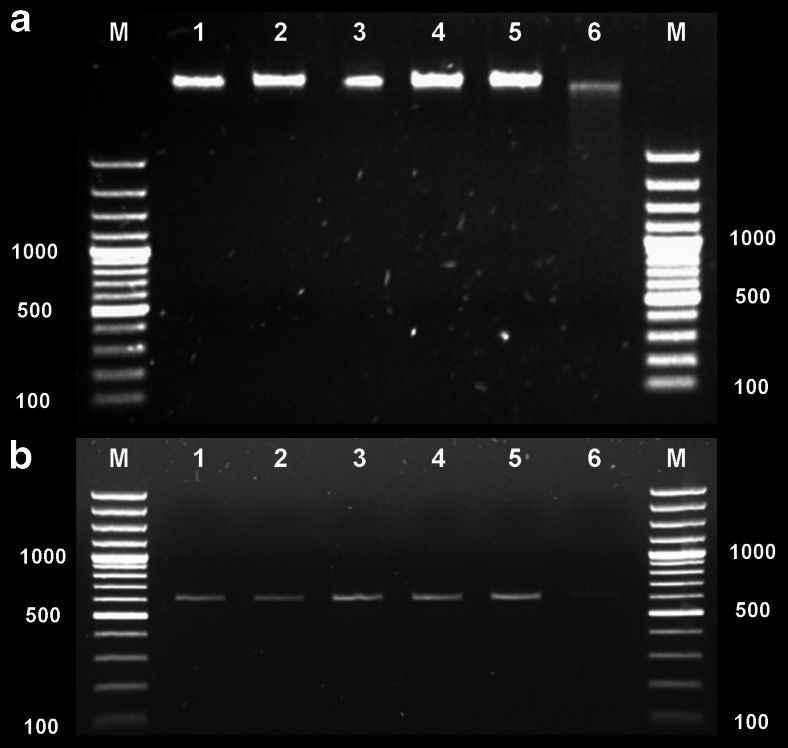
Agarose gel electrophoresis showing genomic DNA and PCR-amplified products of untreated and treated samples. **a** Genomic DNA damage of *S. aureus* Newman. **b** 16S rRNA PCR results. *Lane 1* non-treatment control (bacteria kept in dark); *2* APDT-treated bacteria (1 μM fullerene, 160 J/cm^2^ of white light); *3* APDT-treated bacteria (10 μM fullerene, 160 J/cm^2^ of white light); *4* fullerene-treated bacteria (1 μM, with no illumination); *5* fullerene-treated bacteria (10 μM, with no illumination); *6* TMPyP-treated *S. aureus* (100 μM and 400 J/cm^2^ of 630 nm light)

A pronounced and established fullerene activity is DNA cleavage; thus, *S. aureus*’ genomic DNA integrity was compared before and after fulleropyrrolidine-mediated APDT. Genomic DNA extracts were run on agarose gel electrophoresis as shown in Fig. 5a. DNA samples were quantified by absorbance at 260 nm and adjusted to each lane’s loading. The fullerene-mediated photodynamic treatment did not produce significant genomic DNA cleavage, even in APDT treatment conditions resulting in >6 log reduction in viable counts (Fig. 5a, line 3).

To further assess fulleropyrrolidine effect on genomic DNA, DNA amplifications by PCR was performed as described above. Figure 5b represents PCR-amplified products of untreated and APDT-treated samples run on agarose gel electrophoresis. DNA samples were quantified by absorbance at 260 nm and adjusted to the same loading amount in each lane. PCR results which are more sensitive for detection of small amounts of DNA revealed weaker band intensity only in the case of positive-control sample (TMPyP-treated cells) (Fig. 5b, line 6), thus indicating that fullerene–APDT treatment does not result in damage of DNA.

### Cytotoxicity and phototoxicity of fulleropyrrolidine against eukaryotic cells

**Fig. 6:**
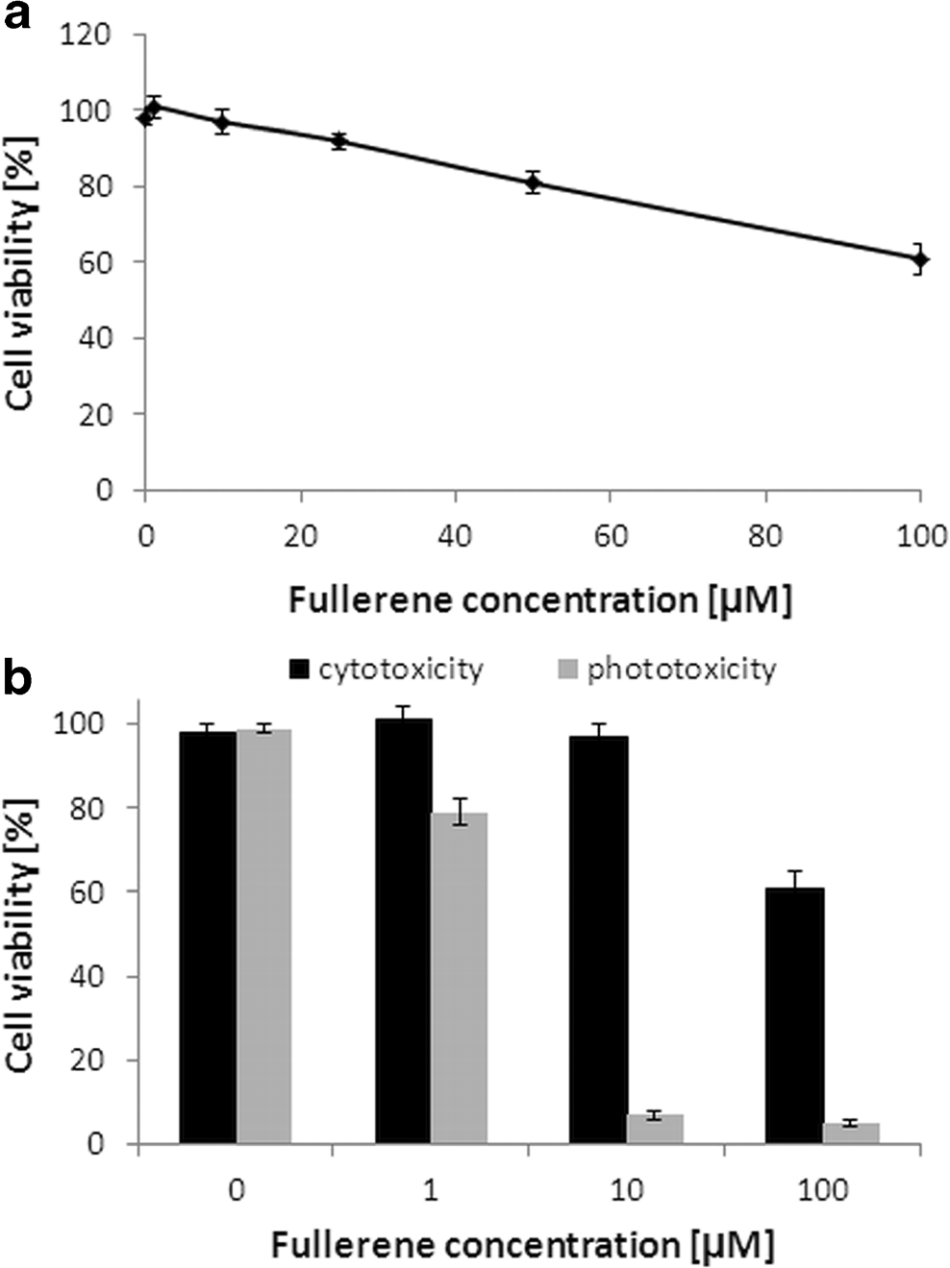
Cytotoxic and phototoxic effect of fulleropyrrolidine toward eukaryotic cells. **a** Toxicity of fulleropyrrolidine after 30-min incubation at 37 °C in the dark. Each point is the mean of three experiments ± SD. **b** Phototoxicity of the sensitizer. Illumination parameters: 10 min, 160 J/cm^2^

The cytotoxic effects of fulleropyrrolidine-mediated APDT in eukaryotic cells was investigated using the MTT assay for different concentrations of the photosensitizers in human dermal keratinocyte (HaCaT). As shown in Fig. 6b, incubation of keratinocytes with fullerene derivative yielded reduced cell viability upon illumination. Incubation of keratinocytes at concentrations up to 100 μM without illumination did not significantly influence cell viability (Fig. 6a). The 50 % effective concentration (EC50) after 30 min of incubation in the dark was not reached at the maximum concentration of 100 μM.

The concentrations of fullerene used in the bacterial and cell toxicity experiments showed concentration-dependent differences upon illumination. Fulleropyrrolidine at a concentration of 1 μM exerted 3.5 log_10_ unit reduction in viable count after illumination, whereas at this concentration, keratinocytes were still viable with and without illumination (Fig. 6).

### In vivo APDT studies

In vivo experiments were performed using the bioluminescent derivative of MRSA strain, Xen31, to analyze if high in vitro effectiveness could be easily translated into in vivo activity.

**Fig. 7:**
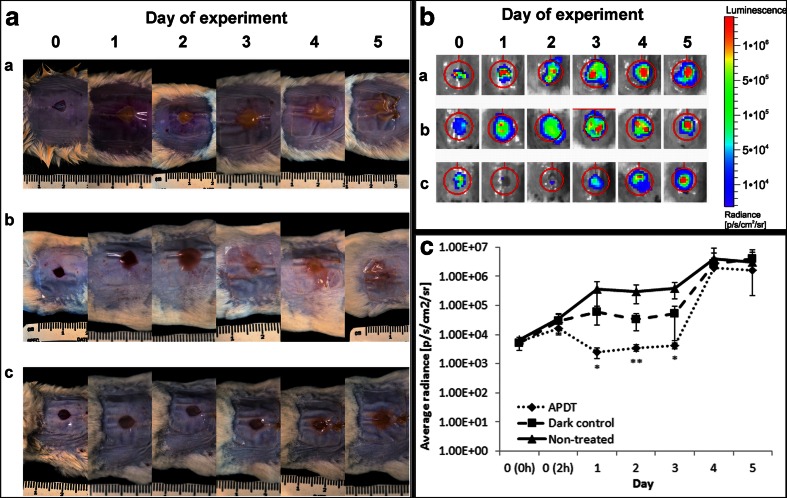
Fulleropyrrolidine-photodynamic therapy of *S. aureus* wound-infected mice. **a** Color photographs of infection sites (wounds created at the mice back) of animals from different experimental groups (*a*, *b*, and *c*) (non-treated, fullerene-treated with no illumination, and APDT-treated animals, respectively). **b** Bioluminescence images of mice infected with *S. aureus*. Non-treated animals (*a*), treated with sensitizer but kept in dark (*b*), and treated after 30 min with fulleropyrrolidine solution and green light (*c*); **c** Quantification of bioluminescence values from the images shown in **b**; *p < 0.05; **p < 0.01 versus non-treated animals

Results for in vivo photodynamic inactivation on mice wounds infected by 5 × 10^6^ CFU of *S. aureus*, with fullerene concentration of 7 × 10^7^ molecules per bacterium, are shown in Fig. 7. In the case of non-treated animals, infection sites were growing systematically from 7.9 × 10^3^ to 3.2 × 10^6^ [hv/s/cm^2^/sr] within 5 days of experiment. The development of *S. aureus* infection sites on mice from dark control group was significantly slower in comparison to non-treated animals (*p* < 0.05). The difference of average radiance between mentioned groups was 1 log_10_ (1.6 × 10^5^ instead of 1.4 × 10^6^ [hv/s/cm^2^/sr]) within first 3 days of experiment but on fourth day got the same level of average radiance as non-treated group (3.8 × 10^6^ [hv/s/cm^2^/sr]). Light control group revealed that infection development trends the same as non-treated control, and hence, for ease of presentation, this group was excluded from Fig. 7. The highest therapeutic effect was observed for the APDT group. The bioluminescent signal increased slightly within the first 2 h post APDT treatment, but 24 h post light administration dramatically decreased and reached background level (3 × 10^3^ [hv/s/cm^2^/sr]). On the fourth day, the bioluminescent signal started to increase, and on fifth day, it was comparable to non-treated and dark control groups.

Daily taken bioluminescence images are shown in Fig. 7b for illustrative purpose. One can observe significantly reduced bioluminescent signal in the APDT group at days 2 and 3 post light irradiation experiment, whereas signal from infected but non-treated wounds increased continuously. There was no bioluminescence detected in not infected mice wounds, indicating no cross-contamination between mice (data not shown).

Bioluminescent signal and the resulting radiance are not sufficient quantifiers to describe infection rate and wound appearance. As shown in Fig. 7a, infected wound on a non-treated mouse started to become moist and yellow starting of the first day of the experiment. Moreover, the surrounding skin was red and swollen. Over the next days, the infection continuously got worse and from day 4 on started to burden also healthy tissues around the wound. In comparison to non-treated mice, infected wound from dark control animals started to be moist 1 day later, and moreover, even at the fifth day, surrounding tissues were not as occupied as in non-treated group. In APDT-treated mice, severe infection was observed at third/fourth day, and bacteria did not burden surrounding healthy skin until fifth day.

## Discussion

Recently, fullerenes, and in particular, functionalized fullerene derivatives, are being intensively studied to determine their biomedical applicability, including potential use in PDT (Mroz et al. 2007c). Fullerenes display several advantages over widely used sensitizers such as porphyrin-based compounds or phenothiazines. These features include high absorption coefficients, photostability, and production of reactive oxygen species via the type I mechanism rather than singlet oxygen (Mroz et al. 2007b). This, compared to photosensitizers that operate via type II process, could be advantageous under some circumstances. Although singlet oxygen is capable of inducing oxidative damage to key cellular constituents, efficient photogeneration of this ROS typically requires relatively high concentration of ground-state molecular oxygen in the location occupied by photosensitizer molecules. It is the consequence of physicochemical processes that are responsible for photosensitized formation of singlet oxygen (Kozinska et al. 2012). On the other hand, oxidizing radicals that are formed via type I photochemical reactions may induce critical damage to photodynamically treated cells and tissues under relatively low oxygen concentration.

The fulleropyrrolidine examined in this study, in aqueous solution, photogenerates different reactive oxygen species with very little or no singlet oxygen. It is in accordance with data by Yamakoshi et al. (Yamakoshi et al. 2003), indicating that photoinduced fullerene derivatives produced type I reactive oxygen species. On the other hand, Mroz et al. (Mroz et al. 2007a) reported that mono-cationic fullerene produced ROS via type I as well as type II mechanism. This apparent discrepancy can be resolved if the generation of ROS photosensitzed by fullerenes is related to specific solvents employed in the experiments performed. It is generally accepted that effective antimicrobial sensitizer should demonstrate high singlet oxygen quantum yields as singlet oxygen is the major cytotoxic species responsible for photoinduced cell killing. For the studied fullerene derivative, we have shown that the photogeneration of singlet oxygen in organic polar solvent as well as in detergent micelles (they form both hydrophobic and hydrophilic microenvironments) is significant, similar to that of rose bengal. The present study indicates that the fullerene photosensitizer localizes mainly in cell envelopes. This can be inferred from the fact that the photodamage of cell membranes is the prime target in the photoinactivation process. Thus, we can assume that the examined fulleropyrrolidine acts mainly via the production of singlet oxygen (type II photooxidation process), although the cytotoxic action of other ROS, including oxidizing radicals generated via type I mechanism, cannot be excluded (as shown in Fig. 2a).

Fulleropyrrolidine iodide salts, in particular mono- and three-cationic fullerene derivatives, are widely studied in the context of antimicrobial PDT (Huang et al. 2010; Lu et al. 2010; Thota et al. 2012). The current study reveals a high in vitro bactericidal effect of mono-cationic fulleropyrrolidine toward gram-positive, gram-negative, and fungal cells. Generally, cationic fullerenes gave high levels of dark toxicity, except for mono-cationic derivatives or di-serinol-functionalized C_60_ which had no dark toxicity (Mroz et al. 2007c). In the case of our sensitizer, typical photodependent toxicity and low dark toxicity toward *S. aureus* cells was revealed at an efficacy higher than described for other mentioned sensitizers at comparable fullerene concentrations. The here studied fulleropyrrolodine iodide salt exerts high bactericidal efficacy primarily against gram-positive bacteria, not surprising considering that most of the sensitizing compounds effectively penetrate gram-positive and fungal cells (Malik et al. 1992). As reported previously, total cationic charge of the sensitizer is extremely important in the case of antimicrobial PDT of gram-negative bacteria characterized with a double-membrane structure that excludes many anionic and uncharged photosensitizing molecules (Malik et al. 1992). Thus, further efforts should be focused on chemical modification of present sensitizer resulting in increased total cationic charge. Such a modification should lead to generation of more effective sensitizer acting toward wide panel of gram-positive as well as gram-negative microbial cells.

To our knowledge, this is the first report in which mechanistic aspects concerning mono-cationic fullerene derivative-mediated PDT were studied. It is assumed that prime target for photodynamic action is the cell membrane envelope. Microbial cells are generally characterized with negative charge; thus, it is thought that cationic sensitizers could easily bind to the outer layers of the cell surface and diffuse to the cell membrane where the photomediated production of ROS can lead to alterations in membrane structure allowing leakage of essential components, loss of membrane integrity, and cell death. Fang et al. (Fang et al. 2007) revealed that bacterial phospholipids can be altered after fullerene exposure depending on bacterial species. In the case of antimicrobial PDT, it has never been reported that mono-cationic fullerene derivative induces cell membrane damage. The results of the present study indicate that structural changes or alterations in the cell membrane of *S. aureus* must occur after photodynamic action as integrity of bacterial envelope decreases significantly. It suggests that cell membrane is the prime target for fullerene-mediated APDT and plays a major role in *S. aureus* damage.

Next, one of the first biological applications of photoactivated fullerenes was to produce cleavage of DNA strands after illumination (Ikeda et al. 2007). Iwamoto et al. (Iwamoto & Yamakoshi 2006) also observed DNA cleavage by functionalized C_60_. This fullerene activity could result in mutagenic effect if present in photodynamic treatment. In fact, solubilized fullerene, when irradiated with visible light, was found to be mutagenic for *Salmonella* strains TA102, TA104, and YG3003 in the presence of rat liver microsomes (Sera et al. 1996). The mutagenicity was elevated in a repair enzyme-deficient mutant of TA102. It was assumed that this mutagenicity results from singlet oxygen action leading to lipid peroxidation of linoleate that causes oxidative DNA damage. Fullerene derivative studied in the present work revealed lack of singlet oxygen production in polar solvents, what probably results in no or undetectable level of DNA damage. It suggests that current sensitizer should not exert mutagenic effect toward both prokaryotic and eukaryotic cells. Obviously, one could claim that few lesions in the DNA is not sufficient to produce a detectable damage by electrophoresis; however, as indicated by positive control (TMPyP-mediated APDT), sensitizers effectively affecting genomic DNA lead to high disruption of DNA integrity that could be easily detected by electrophoresis (Fig. 5a, line 6). Moreover, employed TMPyP-mediated treatment resulted in 4 log reduction in viable counts and not >6 logs, as indicated for fulleropyrrolidine. It suggests that if DNA photocleavage activity would be present in the course of fullerene-mediated APDT, it should easily be detected with the use of agarose gel electrophoresis.

Recent studies conclude that C_60_ itself is non-toxic (Gharbi et al. 2005). In the case of healthy human tissues, it was reported that accumulation and retention time is lower and shorter when comparing to cancer cells (Tabata et al. 1997). Moreover, the sensitizer was excreted without being accumulated in any specific organ. Also Scrivens et al. (Scrivens et al. 1994) demonstrated that fullerene uptake by human keratinocytes required time up to 6 h for 50 % of added fullerene to reach sufficient concentration for effective APDT. It allows easy determination of therapeutic window where the difference between prokaryotic and eukaryotic cell uptake is high enough to reduce the phototoxic effects toward eukaryotic cells, and hence, we selected also only 30 min incubation for the human dermal fibroblast experiments. The short incubation time is valid as long as the wound and exposed skin are subsequently protected from incidental light exposure. Other studies employing human dermal fibroblast showed that dark toxicity of C_60_ exists and it is due to lipid peroxidation (Sayes et al. 2005). In contrast, experiments performed in rats with administration of fullerene by inhalation demonstrated little or no differences in lung toxicity between control and C_60_-treated animals (Sayes et al. 2007). In the case of currently described fullerene derivative, very low dark toxicity was reported toward human keratinocytes, and in the range of studied concentrations, the EC50 was not observed. Nevertheless, photomediated toxicity revealed high eukaryotic cell inactivation, resulting in decreased cell viability of up to 80 % (Fig. 6b). With that result, one could question the suitability of fulleropyrrolidine to serve as an antimicrobial. Nevertheless, as APDT treatment is mainly a localized and time-controlled process; the photoinactivation of eukaryotic cells might be reduced to acceptable level in in vivo experiments. It is worth mentioning that no harmful effect of photoinduced fulleropyrrolidine against healthy mice tissues was reported.

In this report, we describe the mouse model of a skin wound infected with MRSA by using a bioluminescent MRSA derivative of ATCC 33591. Topical application of fulleropyrrolidine iodide salt to the infected wound followed by illumination with green light was carried out. Application of 10^6^ CFU of MRSA onto skin wound and using Tegaderm™ dressing resulted in a stable infection. The results showed that APDT was the most effective when it was carried out 30 min after the application of the bacteria to the surface of the wound. In case of prolonged incubation time in the open wound, no bactericidal effect was reached (data not shown). At 30 min equal to about one doubling time, *S. aureus* cells had not penetrated deep into the wound and remained superficial and additionally, the MRSA biochemistry had not adapted to the new host. While the level of regrowth of *S. aureus* in APDT-treated wounds at the fourth day after treatment was similar in comparison to the control animals, APDT here nevertheless opened a 2-day delay for other therapies or antibodies to be evaluated. Additionally, as indicated by color images of infected sites, even at fourth and fifth day of experiment, the infected site did not demonstrate the same *S. aureus*-burdened as in non-treated animals. It must be emphasized that the only light source that could be used in in vivo studies was lamp-emitting green light (525 ± 15 nm) which is relatively inefficient in fullerene induction. Here comes the comparison of the absorbed photons between the white and the green light source as well as for the experiments with the red light. It suggests that evident therapeutic effect was reached and by improving total cationic charge of the studied sensitizer and by applying white light source in in vivo experiments, one could reach more pronounced therapeutic effect. Additionally, previous studies of Dai et al. employing gram staining of the histological section of the infected skin wound showed that MRSA was localized superficially in the epidermis (Dai et al. 2010). Thus, with the use of green light, one should be able to induce fullerene in the range of bacterial occurrence. Moreover, fullerenes may exert high therapeutic effect in antimicrobial PDT, in which rather superficial infections occur and light does not need to penetrate more than 1 mm (Lu et al. 2010). The case is the low light absorption of fullerene within green range of visible light.

Finally, we report the use of highly effective in vitro, antistaphylococcal photoinducible agent with potential to be used against *E. coli* or *C. albicans* cells. The very first screening tests indicate that the fulleropyrrolidines may be safe and reveal low toxicity against healthy human dermal cells. It acts generally through disrupting of bacterial envelope integrity and not DNA damage assuring its low mutagenicity. In vivo studies indicated that the APDT treatment using fulleropyrrolidine iodide salt is beneficial in the case of *S. aureus*-infected wounds; however, the effect is periodic and followed with bacterial regrowth. Probably, it is due to the usage of light source emitting wavelenghts of 525 ± 15 nm, which corresponds to relatively low absorbance of fullerene within this spectrum. Applying white light illumination, covering whole absorbance spectrum of the sensitizer, would assure higher therapeutic effect.

Next, as mentioned previously by Mizuno et al. (Mizuno et al. 2011), maximizing the number of cationic charges in functionalized fullerenes results in its higher bactericidal activity. Thus, next efforts must be made to synthesize polycationic fullerene derivative and testing its efficacy with the usage of broad-band white light sources.

## References

[CR1] Al-Talib H, Yean CY, Al-Khateeb A, Hassan H, Singh KK, Al-Jashamy K, Ravichandran M (2009). A pentaplex PCR assay for the rapid detection of methicillin-resistant *Staphylococcus aureus* and Panton-Valentine Leucocidin. BMC Microbiol.

[CR2] Castano AP, Demidova TN, Hamblin MR (2004). Mechanisms in photodynamic therapy: part one-photosensitizers, photochemistry and cellular localization. Photodiagnosis Photodyn Ther.

[CR3] Chen CZ, Cooper SL (2002). Interactions between dendrimer biocides and bacterial membranes. Biomaterials.

[CR4] Connell S, Li J, Shi R (2013). Synergistic bactericidal activity between hyperosmotic stress and membrane-disrupting nanoemulsions. J Med Microbiol.

[CR5] Da Ros T, Prato M, Carano M, Ceroni P, Paolucci F, Roffia S (1998). Enhanced acceptor character in fullerene derivatives. Synthesis and electrochemical properties of fulleropyrrolidinium salts. J Am Chem Soc.

[CR6] Da RT, Prato M, Novello F, Maggini M, Banfi E (1996). Easy access to water-soluble fullerene derivatives via 1,3-dipolar cycloadditions of azomethine ylides to C(60). J Org Chem.

[CR7] Dai T, Tegos GP, Lu Z, Huang L, Zhiyentayev T, Franklin MJ, Baer DG, Hamblin MR (2009). Photodynamic therapy for *Acinetobacter baumannii* burn infections in mice. Antimicrob Agents Chemother.

[CR8] Dai T, Tegos GP, Zhiyentayev T, Mylonakis E, Hamblin MR (2010). Photodynamic therapy for methicillin-resistant *Staphylococcus aureus* infection in a mouse skin abrasion model. Lasers Surg Med.

[CR9] Fang J, Lyon DY, Wiesner MR, Dong J, Alvarez PJ (2007). Effect of a fullerene water suspension on bacterial phospholipids and membrane phase behavior. Environ Sci Technol.

[CR10] Francis KP, Yu J, Bellinger-Kawahara C, Joh D, Hawkinson MJ, Xiao G, Purchio TF, Caparon MG, Lipsitch M, Contag PR (2001). Visualizing pneumococcal infections in the lungs of live mice using bioluminescent *Streptococcus pneumoniae* transformed with a novel gram-positive lux transposon. Infect Immun.

[CR11] Gharbi N, Pressac M, Hadchouel M, Szwarc H, Wilson SR, Moussa F (2005). [60]fullerene is a powerful antioxidant in vivo with no acute or subacute toxicity. Nano Lett.

[CR12] Huang L, Terakawa M, Zhiyentayev T, Huang YY, Sawayama Y, Jahnke A, Tegos GP, Wharton T, Hamblin MR (2010). Innovative cationic fullerenes as broad-spectrum light-activated antimicrobials. Nanomedicine.

[CR13] Ikeda A, Doi Y, Hashizume M, Kikuchi J, Konishi T (2007). An extremely effective DNA photocleavage utilizing functionalized liposomes with a fullerene-enriched lipid bilayer. J Am Chem Soc.

[CR14] Isobe H, Nakanishi W, Tomita N, Jinno S, Okayama H, Nakamura E (2006). Gene delivery by aminofullerenes: structural requirements for efficient transfection. Chem Asian J.

[CR15] Iwamoto Y, Yamakoshi Y (2006). A highly water-soluble C60-NVP copolymer: a potential material for photodynamic therapy. Chem Commun (Camb).

[CR16] Jett BD, Hatter KL, Huycke MM, Gilmore MS (1997). Simplified agar plate method for quantifying viable bacteria. Biotechniques.

[CR17] Kozinska A, Oles T, Sarna T (2012). Photoactivation and detection of photoexcited molecules and photochemical products. Isr J Chem.

[CR18] Kroto HW, Heath JR, O'Brien SC, Curl RF, Smalley RE (1985). C60: Buckminsterfullerene. Nature.

[CR19] Lilge L, Tierney K, Nussbaum E (2000). Low-level laser therapy for wound healing: feasibility of wound dressing transillumination. J Clin Laser Med Surg.

[CR20] Lu Z, Dai T, Huang L, Kurup DB, Tegos GP, Jahnke A, Wharton T, Hamblin MR (2010). Photodynamic therapy with a cationic functionalized fullerene rescues mice from fatal wound infections. Nanomedicine (Lond).

[CR21] Malik Z, Ladan H, Nitzan Y (1992). Photodynamic inactivation of gram-negative bacteria: problems and possible solutions. J Photochem Photobiol B.

[CR22] Marchesan S, Da RT, Spalluto G, Balzarini J, Prato M (2005). Anti-HIV properties of cationic fullerene derivatives. Bioorg Med Chem Lett.

[CR23] Mizuno K, Zhiyentayev T, Huang L, Khalil S, Nasim F, Tegos GP, Gali H, Jahnke A, Wharton T, Hamblin MR (2011). Antimicrobial photodynamic therapy with functionalized fullerenes: quantitative structure-activity relationships. J Nanomed Nanotechnol.

[CR24] Mroz P, Pawlak A, Satti M, Lee H, Wharton T, Gali H, Sarna T, Hamblin MR (2007). Functionalized fullerenes mediate photodynamic killing of cancer cells: type I versus type II photochemical mechanism. Free Radic Biol Med.

[CR25] Mroz P, Pawlak A, Satti M, Lee H, Wharton T, Gali H, Sarna T, Hamblin MR (2007). Functionalized fullerenes mediate photodynamic killing of cancer cells: type I versus type II photochemical mechanism. Free Radic Biol Med.

[CR26] Mroz P, Tegos GP, Gali H, Wharton T, Sarna T, Hamblin MR (2007). Photodynamic therapy with fullerenes. Photochem Photobiol Sci.

[CR27] Sayes CM, Gobin AM, Ausman KD, Mendez J, West JL, Colvin VL (2005). Nano-C60 cytotoxicity is due to lipid peroxidation. Biomaterials.

[CR28] Sayes CM, Marchione AA, Reed KL, Warheit DB (2007). Comparative pulmonary toxicity assessments of C60 water suspensions in rats: few differences in fullerene toxicity in vivo in contrast to in vitro profiles. Nano Lett.

[CR29] Scrivens WA, Tour JM, Creek KE, Pirisi L (1994). Synthesis of C-14-labeled C-60, its suspension in water, and its uptake by human keratinocytes. J Am Chem Soc.

[CR30] Sera N, Tokiwa H, Miyata N (1996). Mutagenicity of the fullerene C60-generated singlet oxygen dependent formation of lipid peroxides. Carcinogenesis.

[CR31] Tabata Y, Murakami Y, Ikada Y (1997). Photodynamic effect of polyethylene glycol-modified fullerene on tumor. Jpn J Cancer Res.

[CR32] Tang YJ, Ashcroft JM, Chen D, Min G, Kim CH, Murkhejee B, Larabell C, Keasling JD, Chen FF (2007). Charge-associated effects of fullerene derivatives on microbial structural integrity and central metabolism. Nano Lett.

[CR33] Tegos GP, Demidova TN, Arcila-Lopez D, Lee H, Wharton T, Gali H, Hamblin MR (2005). Cationic fullerenes are effective and selective antimicrobial photosensitizers. Chem Biol.

[CR34] Thota S, Wang M, Jeon S, Maragani S, Hamblin MR, Chiang LY (2012). Synthesis and characterization of positively charged pentacationic [60]fullerene monoadducts for antimicrobial photodynamic inactivation. Molecules.

[CR35] Yamakoshi Y, Sueyoshi S, Miyata N (1999) [Biological activity of photoexcited fullerene]. Kokuritsu Iyakuhin Shokuhin Eisei Kenkyusho Hokoku 117:50–60 10859936

[CR36] Yamakoshi Y, Umezawa N, Ryu A, Arakane K, Miyata N, Goda Y, Masumizu T, Nagano T (2003) Active oxygen species generated from photoexcited fullerene (C60) as potential medicines: O_2_^-*^ versus ^1^O_2_. J Am Chem Soc 125:12803–12809 10.1021/ja0355574 14558828

[CR37] Zakharian TY, Seryshev A, Sitharaman B, Gilbert BE, Knight V, Wilson LJ (2005). A fullerene-paclitaxel chemotherapeutic: synthesis, characterization, and study of biological activity in tissue culture. J Am Chem Soc.

